# P-Glycoprotein: One Mechanism, Many Tasks and the Consequences for Pharmacotherapy of Cancers

**DOI:** 10.3389/fonc.2020.576559

**Published:** 2020-10-26

**Authors:** Anna Seelig

**Affiliations:** Biozentrum, University of Basel, Basel, Switzerland

**Keywords:** P-glycoprotein inhibition, P-glycoprotein upregulation, P-glycoprotein substrate binding, membrane-mediated binding, pattern recognition 1, cancer metabolism, immune surveillance, immune suppression

## Abstract

P-glycoprotein or multidrug resistance protein (MDR1) is an adenosine triphosphate (ATP) binding cassette transporter (ABCB1) intensely investigated because it is an obstacle to successful pharmacotherapy of cancers. P-glycoprotein prevents cellular uptake of a large number of structurally and functionally diverse compounds, including most cancer therapeutics and in this way causes multidrug resistance (MDR). To overcome MDR, and thus improve cancer treatment, an understanding of P-glycoprotein inhibition at the molecular level is required. With this goal in mind, we propose rules that predict whether a compound is a modulator, substrate, inhibitor, or inducer of P-glycoprotein. This new set of rules is derived from a quantitative analysis of the drug binding and transport properties of P-glycoprotein. We further discuss the role of P-glycoprotein in immune surveillance and cell metabolism. Finally, the predictive power of the proposed rules is demonstrated with a set of FDA approved drugs which have been repurposed for cancer therapy.

## Introduction

“The evolution of multidrug resistance (MDR) remains one of the major barriers to a control or cure of cancer” (1). Although, MDR is multifactorial in etiology it is essentially associated with overexpression of ATP binding cassette (ABC) transporters (2, 3). ATP-binding cassette (ABC) transporters constitute a ubiquitous superfamily of integral membrane proteins, divided into seven subfamilies (ABCA to ABCG). The functional unit of ABC transporters consists of two nucleotide binding domains that hydrolyze ATP in a magnesium dependent manner, and two transmembrane domains (4, 5) that bind and translocate a large number of exogenous and endogenous compounds across membranes. The best investigated ABC transporter is P-glycoprotein (Pgp/MDR1/ABCB1) (molecular mass, MM = 170 kDa). It is encoded by the multidrug resistance gene (*mdr*/*MDR*) and was originally identified in colchicine resistant Chinese hamster ovary cells by Juliano and Ling (6). Amplification of the *MDR* genes in mammalian cell lines by a single agent (e.g., colchicine) induced cross-resistance to a remarkably wide range of compounds with no obvious structural or functional similarities (7). The *mdr*/*MDR* gene has three isoforms in rodents (*mdr1a, mdr1b*, and *mdr2*) and two isoforms in humans (*MDR1* and *MDR2*). MDR1 functions as transporter of amphiphilic compounds including drugs and certain lipids (8), whereas MDR2 is primarily a lipid transporter (9, 10). The isoforms exhibit partially overlapping substrate specificity.

The multidrug resistance proteins (MRPs, ABCCs) (11–13) and the breast cancer resistance protein (BCRP, ABCG2) (14) are further ABC family members that contribute to MDR [see e.g., (15)]. The three transporters also exhibit partially overlapping substrate specificity with Pgp and recognize compounds based on related physical-chemical principles (16–18). Such redundancies are typical for important cellular defense mechanisms.

Many tumor types overexpress Pgp (19) [for review see (20–23)] which prevents cancer drugs from reaching their cellular targets. Conventional cytotoxic drugs that interfere with DNA replication pathways, killing the rapidly dividing cancer cells (24) are particularly prone to extrusion by Pgp. But also newer agents that block the growth and spread of cancer cells by targeting specific molecular pathways (24), generally interact with Pgp. To overcome MDR in cancer chemotherapy it therefore seemed auspicious to search for appropriate Pgp inhibitors.

Numerous compounds were positively tested as Pgp inhibitors in cellular assays. One of the most tested first generation inhibitors is verapamil, a calcium channel antagonist, used as a racemic mixture in the nanomolar concentration range for treatment of cardiovascular diseases (25). As Pgp is not enantioselective, the less toxic enantiomer, R-verapamil, was chosen, but nevertheless exhibited significant side effects, because micromolar concentrations are required for Pgp inhibition (26). Less toxic and more efficient second, third and fourth generation inhibitors were developed (27, 28), but despite these efforts, the overall approach failed in clinical settings (23, 29). Although, the reasons for failure are multifaceted, the “dose limiting toxicity and the lack of specificity of Pgp inhibitors” (3) were considered as the key factors. Additional, less well understood issues related to the role of Pgp in immunosurveillance and metabolism are emerging.

In the following we discuss how and where Pgp captures and releases its substrates, recapitulate the consequences of Pgp inhibition in absorption and excretion, and give some insight into the role of Pgp in immunosurveillance and metabolism of cancers. Based on this analysis, we discuss a set of FDA approved drugs, previously repurposed for cancer treatment (30) with respect to their interaction with Pgp.

## The Floppase Model and the Consequences for Drug Pgp Interactions

The key question for understanding Pgp—drug interactions is in which environment Pgp captures its substrates. This is important because, the forces driving drug binding to Pgp differ distinctly depending on whether binding takes place in the aqueous phase or in the lipid phase as outlined below.

In 1992 Gottesman and Higgins (31) discussed two possible models for Pgp function, the pump model and the “flippase” model. The pump model assumes that drugs interact with Pgp in the cytoplasmic aqueous phase, are then pumped across the lipid bilayer membrane, and are released directly into the extracellular aqueous phase. The “flippase” model assumes that drugs first partition into the lipid membrane and then interact with the transmembrane part of Pgp that “flips” the drug from the cytoplasmic to the outer leaflet. From the outer leaflet drugs either diffuse into the extracellular aqueous phase, or flip back to the cytoplasmic leaflet, where they are recaptured. More recently, the movement from the inner to the outer leaflet was defined as flopping, and the inverse movement as flipping. For clarity, we therefore address the model (31) as floppase model in the following.

The floppase model was essentially based on experiments showing that drug binding to Pgp occurs in the cytoplasmic membrane leaflet (32). An unambiguous proof of the floppase activity of Pgp [described as a solvation exchange mechanism (33, 34)], was provided by Omote and Al-Shawi (35). They investigated the transport activity of Pgp proteoliposomes (i.e., Pgp reconstituted in lipid vesicles) using permanently charged spin-labeled verapamil that cannot passively diffuse across the membrane. Labeled verapamil was added to the outside of the vesicles, then partitioned into the outer leaflet of the lipid bilayer, where it was captured by Pgp and was “transported” to the inner leaflet with a turn-over number of 5.8/s. “Transport” lead to a 10-fold accumulation of labeled verapamil in the inner membrane leaflet of the vesicle. Due to the permanent charge on verapamil a high positive surface potential developed, most likely preventing full transport to the inner leaflet. It should be noted that due to the orientation of Pgp in vesicles, transport from outer to the inner leaflet is observed, whereas in cells transport works from the inner to outer leaflet. In the case of amphiphilic drugs (that are generally not permanently charged) high Pgp activity leads to continuous flopping, concomitant expansion of the extracellular membrane leaflet and eventually to membrane budding. This phenomenon plays an important role in immunostimulation (3).

The X-ray structures of the nucleotide free (apo) Pgp (36, 37), showing a wide opening toward the cytoplasm combined with the simple and thus appealing alternate access model (38), led to a paradigm shift, restoring the pump model [see e.g., (39)]. Pgp-substrate binding is thus currently mostly assumed to take place in the cytoplasmic, aqueous environment and consequently, to be driven by hydrophobic interactions between the protein and the substrate. Because of its relative simplicity, this approach seems particularly attractive for molecular modeling.

At first sight, both models seem to be supported by experimental evidences. However, it should be noted that the key requirement for crystallization is to immobilize the protein. Although, precious information is gained from protein structures, obtained by X-ray crystallography, they are not necessarily functional (4, 40). Nevertheless, much research has focused on the properties of the open cleft, thereby neglecting the lipid phase.

In contrast, we provided strong quantitative support for the flopping model by Higgins and Gottesman (31). Drugs interacting with Pgp are all amphiphilic, either electrically neutral, or cationic. They orient with their polar part toward the polar head group region of lipids and with their hydrophobic part toward the hydrophobic fatty acyl chain region. A “turn-around,” flipping or flopping movement of an amphiphilic molecule moving from one leaflet to the other is therefore required. Moreover, amphiphilic drugs partition avidly into lipid membranes. The concentrations of drugs in the lipid phase are therefore orders of magnitude higher than in the aqueous phase, as demonstrated by surface activity and isothermal titration calorimetry measurements (ITC) [e.g., (41)].

**Figure 1:**
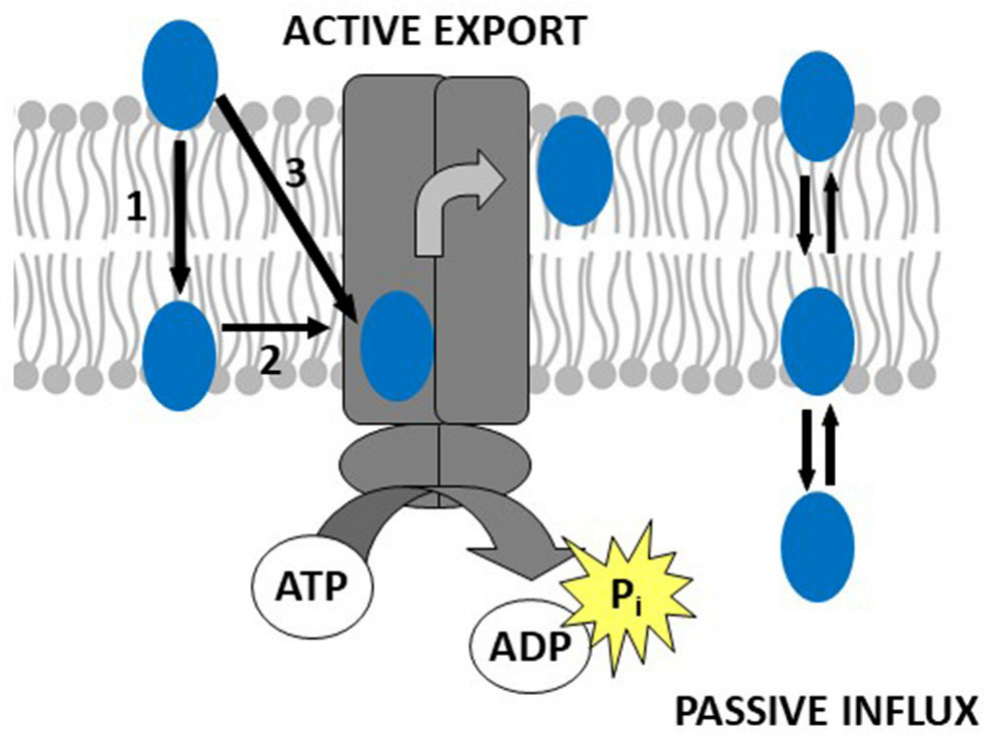
Drug binding to Pgp follows a two-step binding process (left-hand side): a *lipid water partitioning step* described the free energy of lipid-water partitioning $\left(\Delta G_{lw}^{0}\right)$ or “binding affinity” of the drug to the lipid membrane (1) and a *transporter-lipid binding step* with the free energy of transporter-lipid binding $\left(\Delta G_{tl}^{0}\right)$ or “binding affinity” of the drug to the transporter within the lipid phase (2). The overall *transporter-water binding step* described by the free energy of transporter-water binding $\left(\Delta G_{tw}^{0}\right)$ can then be expressed as the sum of the two individual steps (3). The parameters $\left(\Delta G_{tw}^{0}\right)$ (3) and $\left(\Delta G_{lw}^{0}\right)$ (1) are directly measurable, and the parameter$\left(\Delta G_{tl}^{0}\right)$ (2) is determined as the difference of the two [(3)–(1) = (2)]. This approach allows to quantitatively assess the affinity of drugs to Pgp in the lipid membrane. At the right-hand side, we show a molecule that escapes the transporter by passive diffusion.

Thus, drug binding to Pgp is best described as a two-step binding process, starting with a *lipid-water partitioning step* of the drug (characterized by a free energy of lipid-water partitioning), followed by a *transporter-lipid binding step* of the drug, (characterized by a free energy of transporter-lipid binding). The overall *transporter-water binding step* (described by the free energy of transporter-water binding) can then be expressed as the sum of the free energy of lipid-water partitioning and the free energy of the drug binding to Pgp in the lipid membrane. The free energy of transporter-water binding and the free energy of lipid-water partitioning are experimentally accessible, but not the free energy of the drug binding proper. However, the latter can be determined as the difference of the two measurable free energies (Figure 1) (for details see legend to Figure 1).

The movement of a drug from the aqueous phase into the lipid membrane is the consequence of *hydrophobic interactions*, caused by the entropy gain from the release of bound water molecules as the drugs enter the membrane. To capture an amphiphilic, hydrophobic molecule, immersed in the very hydrophobic lipid environment, hydrophobic interactions would not help. In the following we will show that Pgp captures drugs by a different type of interactions.

Thus, we prove the validity of the floppase model (31) and quantify for the first time the affinity of drugs to Pgp in the lipid membrane (42–44) as suggested earlier (45).

### The Forces Governing Drug Capture by Pgp From the Membrane

**Figure 2:**
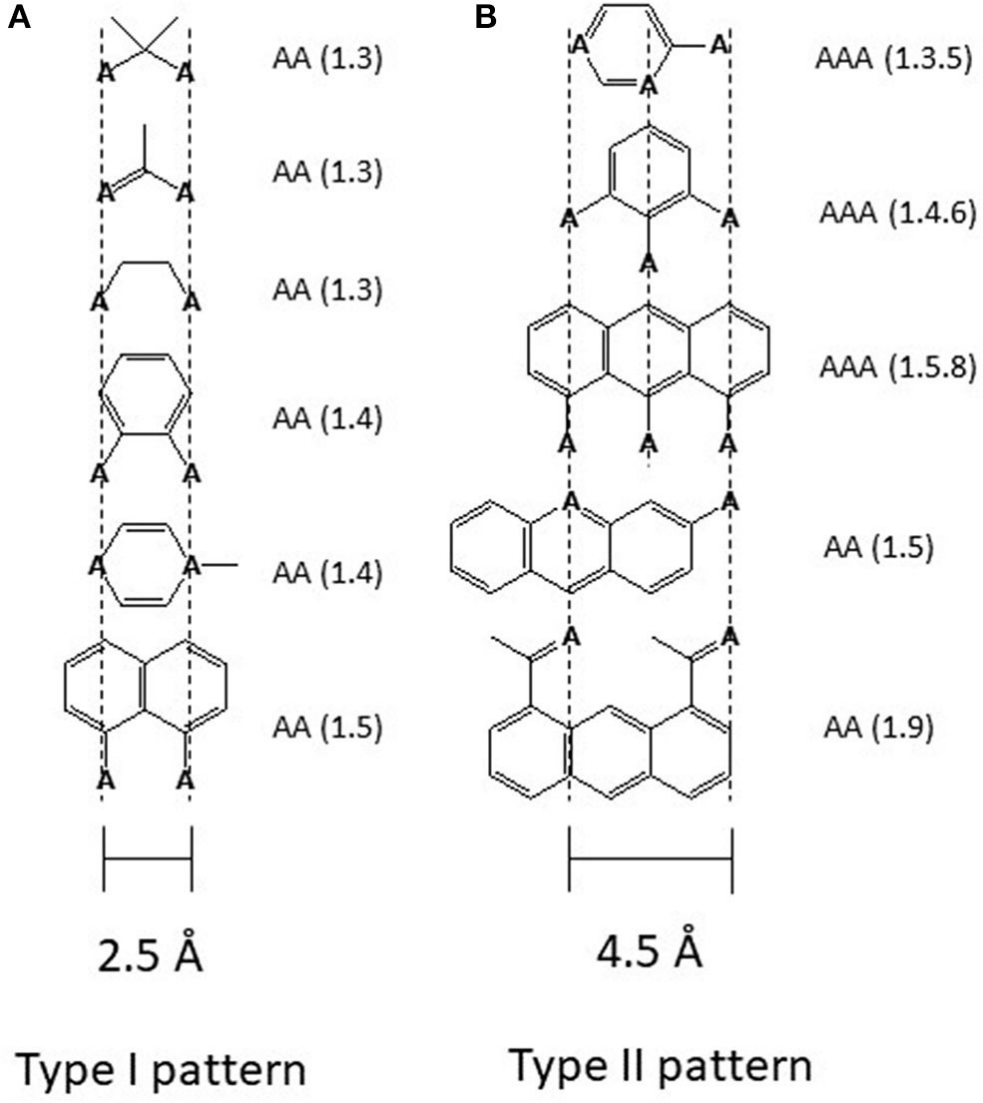
Electron donor or hydrogen bond acceptor patterns observed in P-glycoprotein substrates. **(A)** Patterns formed by electron donor pairs with a spatial separation of 2.5 ± 0.3 Å (type I unit). **(B)** Patterns formed either by three electron donor groups with a spatial separation of the outer two electron donor groups of 4.6 ± 0.6 Å, or by two electron donor groups with a spatial separation of 4.6 ± 0.6 Å (type II unit). “A” denotes a hydrogen bond acceptor group (electron donor group) and the numbers in brackets indicate the first and the nth atom with a free electron pair (46). The relevant hydrogen bond acceptor groups are >C=O (carbonyl group), -O- (ether groups), -NR_3_, -N=, -S-, R-F, >C(C_6_H_5_). All molecules that contain at least one type I or one type II unit were found to interact with Pgp substrates. Molecules that contain a type II unit seem to be in addition, inducers of Pgp over-expression (46), possibly via the pregnane X receptor pathway (PXR) (47). Groups that do *not* interact with Pgp are -OH, -NH_2_, or >NH, however, since they reduce passive diffusion (48) they may be present in substrates.

Drug binding to the transporter can be approached in two ways either by first looking at the transporter or by first looking at the captured molecules. In the case of Pgp the second approach was particularly revealing. Searching for recurrent structural elements in hundreds of Pgp substrates showed that all compounds interacting with Pgp exhibit at least one pattern formed from electron donor groups or π-electron rings in specific distances from each (i.e., hydrogen bond acceptor groups, HBA's) (Figure 2) (18, 46, 49, 50). Because of the low dielectric constant of the lipid phase (ε ~ 2) compared to the aqueous phase (ε ~ 80), electrostatic interactions are up to 40-fold higher in the membrane interior than in the aqueous phase which is favorable for binding of drugs in the membrane and release at the lipid-water interface.

**Figure 3:**
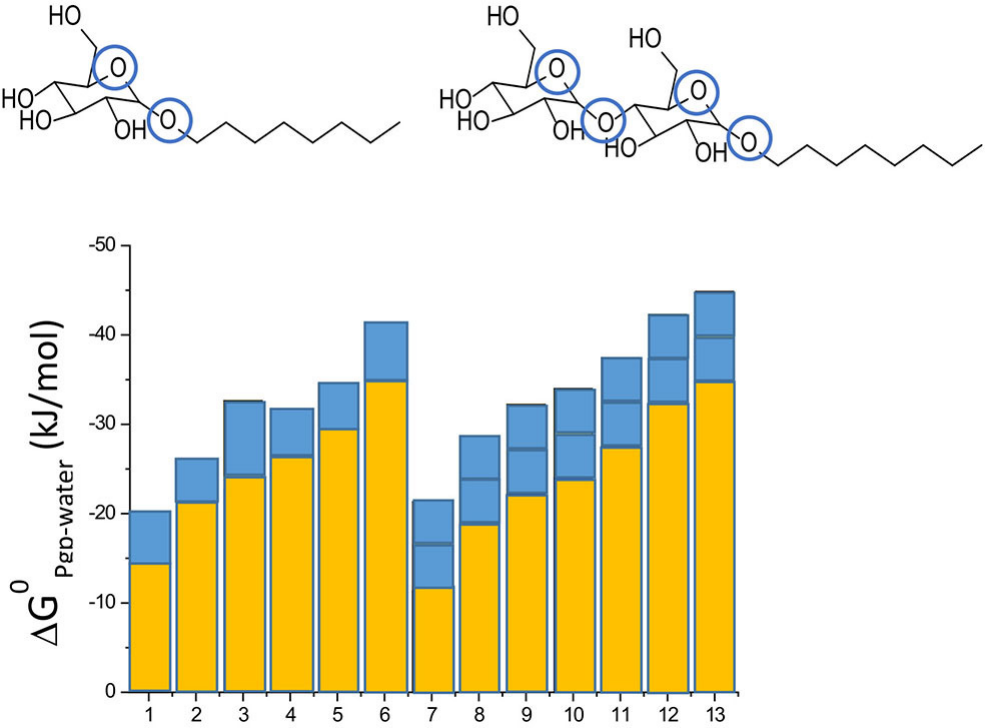
Modular binding principle. The free energies of lipid-water partitioning (yellow) and the free energies of transporter-lipid binding (blue) of n-alkyl-β-D-glucopyranosides and n-alkyl-β-D-maltopyranosides are plotted vs. the alkyl chain lengths: C_6_-gluc (1), C_7_-gluc (2), C_8_-gluc (3), C_9_-gluc (4), C_10_-gluc (5), C_12_-gluc (6), C_6_-malt (7), C_8_-malt (8), C_9_-malt (9), C_10_-malt (10), C_11_- malt (11), C_12_-malt (12), C_13_-malt (13). The suffix n indicates the number of carbon atoms. All the compounds are allocrites for Pgp (43). The binding affinity to maltosides with two type I patterns to Pgp in the lipid membrane is twice as strong as the binding affinity of glucosides with one type II pattern (shown in blue). The binding affinity of the hydrophobic anchors to the membrane increases with the length n shown in yellow). The binding affinity of the compounds from water to the transporter is the sum of the two.

Not only drugs, but also many other endogenous compounds, such as steroid hormones, and exogenous compounds, such as detergents, carry recognition patterns for Pgp. Notably, detergents are bound and flopped by Pgp at concentrations much below those affecting the lipid membrane packing density (43, 51). As detergents are available with varying numbers of hydrophobic methylene, or hydrogen bond acceptors groups, respectively, they are particularly valuable for systematic investigations of compound binding and transport by Pgp. Using such detergents, we found that addition of a methylene group to a compound enhances membrane partitioning and thus also binding to the transporter with a gain in free energy of ΔG_CH2_ ≈ −3 kJ/mol (43, 44), which corresponds to an increase in the binding constant by a factor of ~ 6. Conversely, addition of an ethoxyl group (i.e., a HBA group) is unfavorable for membrane partitioning, however, it enhances binding of the molecule to Pgp within the membrane by ΔG_HBA_ ≈ −2.5 to −4 kJ/mol (depending on the location of the HBA group within the lipid membrane relative to the lipid-water interface) (42–44) (Figure 3). Drug binding to Pgp thus works on a modular basis (Figure 3).

Looking at the transporter, revealed two binding locations, simultaneously accommodating at least two identical (52) or also non-identical molecules (53). The observation of two molecules bound to Pgp at high, inhibitory concentrations was later also observed by other approaches [e.g., (42, 54, 55)].

Scrutinizing the potential translocation pathways for possible interaction sites with the HBAs and π-electron rings in drugs, we found ample hydrogen bond donor groups (HBD) and π-electron rings (e.g., phenyl or tryptophan rings) that could form hydrogen bonds, π-π stacking, and π-cation interactions with drugs (17, 18). Transport of substrate molecules along these groups in the transmembrane helices of Pgp is most likely a stochastic process [for details see (40, 44)]. This assumption is consistent with the finding that the exchange of single, aromatic groups for cysteine in the drug binding region had little effect on drug binding, which was explained with the redundancy of binding sites (56). As cysteine residues exhibit a hydrogen bond acceptor group they may mechanistically even substitute for aromatic residues (18).

Thus, we demonstrated that Pgp captures drugs within the lipid membrane via weak electrostatic interactions between HBAs, including phenyl rings and tryptophanes (i.e., π-electron donor systems), in drugs and HBDs (i.e., π-electron acceptor systems) in the transmembrane region of Pgp. Two or three HBAs are arranged in specific patterns forming type I or type II units, respectively. Minimally one such pattern is required for transport by Pgp, but several patterns can be present in one drug molecule. These weak electrostatic interactions are ideal for attracting drugs in the lipid environment and for releasing them as soon as water is available (44).

## What Defines a Pgp Modulator, Inhibitor, Substrate, Inducer, and Allocrite?

The Pgp ATPase activity assay provides the most direct measurement of *effective* drug transport by Pgp (26, 57–59). For every drug transported at least one ATP molecule is hydrolyzed (58). Pgp exhibits basal activity with a turnover number around 1/s (57). If measured as a function of concentration of small and medium size molecules, such as verapamil (MM = 454.6 g/mol), the Pgp ATPase activity first increases, reaches a maximum at intermediate concentrations (for verapamil at 10 μM), and decreases again at higher concentrations, yielding bell-shaped activity curves as observed in classical ATPase assays as well as in a coupled enzyme assay that allows for ATP regeneration (16). It is assumed that one molecule per Pgp is bound in the rising branch, and more than one in the falling branch. Molecules are still transported if more than one molecule is bound to the transporter. The effect of two molecules simultaneously bound to the transporter was elegantly demonstrated by linking two quinines, which as a monomer shows a high, and as a dimer a strongly reduced ATPase activity (60). For drugs the rate of transport decreases exponentially with increasing affinity to the transporter (45, 61). Thereby, transport (flopping) and release of a drug are most likely the rate limiting steps in the transport cycle of Pgp.

**Pgp Modulators** are compounds that are rather small (MM < 450 g/mol) and carry at least two HBAs thus one type I or type II pattern to interact with Pgp. Depending on the concentration applied they either enhance or reduce the Pgp ATPase activity, i.e., modulate it. As shown below modulators show no net transport by Pgp.

**Pgp Inhibitors** are compounds that slow down the rate of Pgp ATPase activity and transport. Thus, modulators applied at high concentrations, in the falling branch of the ATPase activity curve act as inhibitors [e.g., verapamil at 50 μM (57)]. If applied together with further compounds interacting with Pgp, much lower concentrations can lead to inhibition. As Pgp can accommodate two or more than two non-identical molecules per transport cycle, Pgp inhibition often occurs unintendedly, if more than one drug is applied, a phenomenon dubbed **drug-drug interactions**.

Generally, the inhibitory power of a compound can be enhanced by either strengthening the affinity of the drug to the membrane, by making the molecule more hydrophobic, e.g., by increasing the number of methylene groups (as observed in third and fourth generation inhibitors), or by strengthening the affinity of the drug to the transporter (by increasing the number of hydrogen bond acceptor groups), or both. Cyclosporine A is a classic example with many hydrogen bond acceptor groups that is more efficient than verapamil as inhibitor [e.g., (51)]. Increasing the inhibitory power of drugs with increasing number of hydrogen bond acceptor groups has been repeatedly demonstrated (42, 45, 62).

**Substrates** are generally assessed by bidirectional transport assays [e.g., (63)] that reveal *apparent* or *net* (not effective) transport. A **substrate** is therefore defined as a compound that shows *net efflux* (i.e., higher active efflux by Pgp than passive influx) in a transport assay. We will show below that **substrates** can be defined as molecules that carry at least on hydrogen bond acceptor pattern and exhibit a MM > 450 g/mol.

*Effective* and *net* transport are practically identical if passive diffusion (or passive flipping) is much slower than active flopping or transport. This is true for zwitterionic phosphatidylcholine (PC) lipids. The turnover number for PC lipids in pure lipid vesicles (without transporters) was assessed as 1/2.4 min for flipping and as 1/4.2 min for flopping (64). A lipid floppase such as MDR2 with a turnover number around 1/s is thus able to maintain the asymmetry of a biological membrane with palmitoyl-oleoyl-phosphatidylcholine, POPC (MM = 760.1 g/mol) exclusively in the outer leaflet of human bilayer membranes.

Vinblastine, a cytotoxic drug with a similar molecular mass (MM = 811 g/mol) diffuses or flips however, more rapidly than PC lipids (57), because it is almost non-charged at neutral pH. Smaller drugs (i.e., modulators) diffuse rapidly, and can escape the transporter to a large extent. Thus, small *modulators* reach the cytoplasm, despite being transported more rapidly than larger compounds.

**Figure 4:**
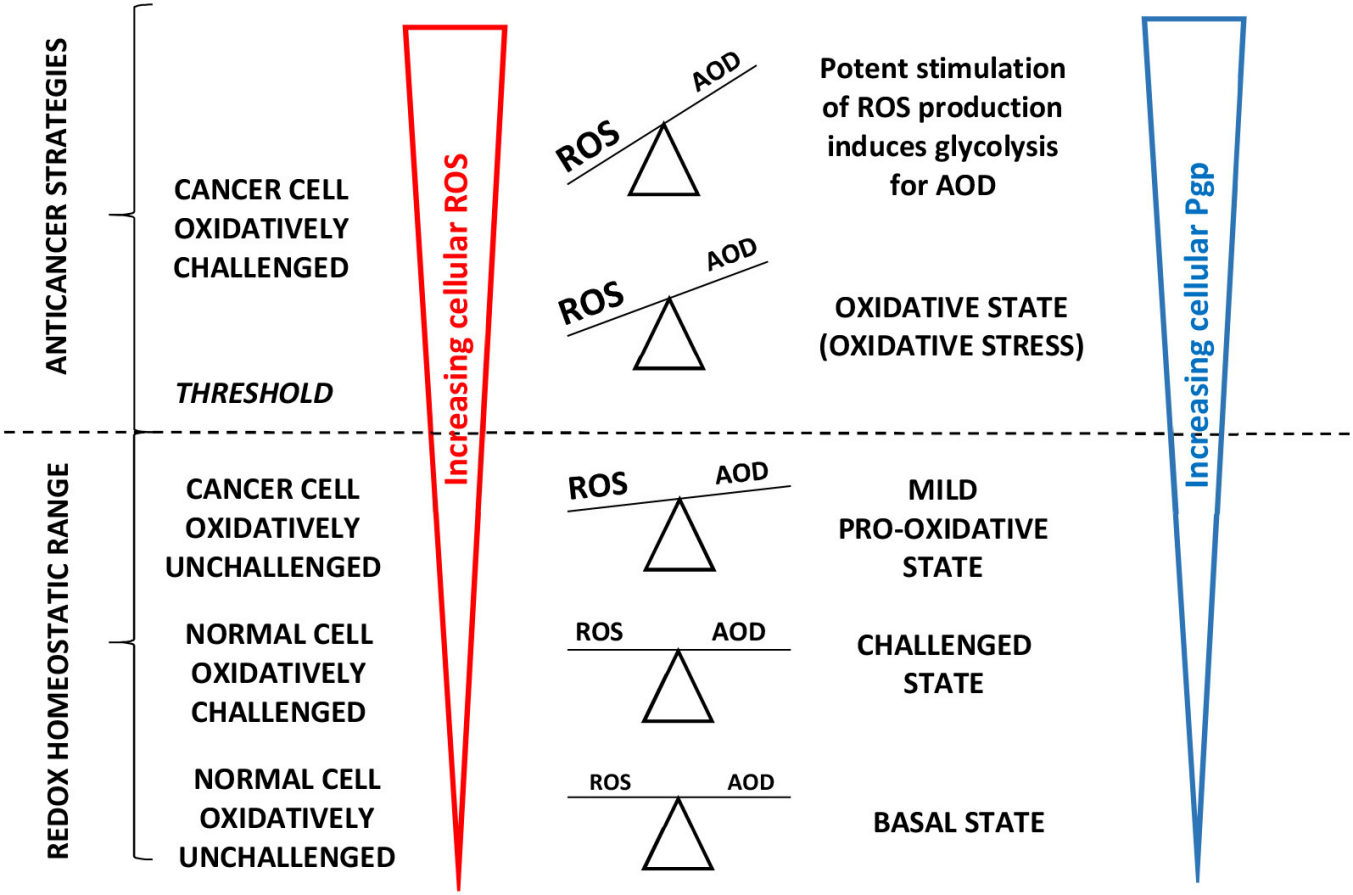
Adapted from Vucetic et al. (66): “The influence of ROS increases with cancer progression and is tightly linked to Pgp expression. Once formed, cancer progression seems to be further stimulated by a mild pro-oxidative state, due to intensified metabolism and ROS-producing foci. Importantly, this state is still maintained within the “redox homeostatic range” thanks to strongly upregulated AOD of cancer cells. However, due to maximized AOD, cancer cells do not support further increase in ROS levels and thus cross the threshold into the state of “oxidative stress.” If ROS levels increase further (e.g., due to chemotherapy), the only way for cancer cells to prevent further damage is by decreasing ROS production via cell-cycle arrest to repair damage and prevent cell death (cytostatic effects of ROS).” Cells react to ROS by upregulating cellular antioxidant defense (AOD) mechanisms and in parallel induce *mdr1b* mRNA and Pgp overexpression (67). “However, if ROS burst induces irreversible damage and/or there is not enough components required for repair systems (e.g., glutathione), cancer cells experience programmed cell death or necrosis (cytotoxic effects of ROS) (66)”.

To disentangle the parameters affecting *net* transport of a drug, we assessed the rate of *effective* drug transport by measuring the Pgp-ATPase activity and calculated the passive flux through the lipid membrane, as it is too fast to be measured for most drugs with the current methods (65). Passive flux decreases exponentially with the cross-sectional area of the drug molecule as well as with the packing density of the lipid membrane (65). The rate of drug diffusion can thus vary by orders of magnitude, depending on the cross-sectional area of the molecule and the membrane packing density. The rate of Pgp-mediated active transport also varies, decreasing exponentially with increasing drug affinity to Pgp. If passive diffusion and active transport are plotted vs. the size of drugs, it becomes evident that the variation in passive diffusion is much higher, than the variation in active transport [Figure 4 in (65)].

Thus, whether or not a drug can be classified as substrate, depends on the cross-sectional area of the drug, as well as on the lipid composition that defines the lipid lateral packing density (65, 68). Notably, the packing density changes with the lipid composition of the membrane and increases e.g., with the cholesterol content, which generally increases with age. If we assume a lateral packing density for biological membranes at T ≈ 37°C of π_M_ = 30 mN/m, the critical cross-sectional area above which a compound becomes a “substrate” for Pgp was assessed as A_D_ ≥ 70 Å^2^ by a theoretical (65) and a phenomenological approach (69). This cross sectional area can be approximated with a molecular mass MM > 450 g/mol.

**Pgp inducers** carry at least one type II pattern (Figure 2). Type II patterns are particularly abundant in Pgp cytotoxic cancer drugs (46). If inducers are small they act as modulators. If they are large they are “substrates,” and if they carry many patterns they inhibit Pgp ATPase activity and transport already at low concentrations and act as inhibitors.

Compounds carrying type II patterns have the ability to induce Pgp expression via nuclear receptors such as the pregnane X receptor (PXR), a ligand-activated nuclear receptor (NR) (70) and the signaling the c-Jun N-terminal kinase (JNK)/AKT/NF-κB) pathway. Thereby AKT stands for protein kinase B (PKB). Examples are rifampine, hyperforin (47) and deoxynivalenol (71), all carrying type II patterns.

In this context it is interesting to note that ferulic acid, which is strongly acidic (pK_a_ 3.27) and is no Pgp allocrite, reverses Pgp expression and MDR via inhibiting the phosphatidylinositol 3-kinase (PI3K)/AKT/NF-κB signaling pathway (72).

To rejoin all compounds (modulators, inhibitors, substrates and inducers) interacting with Pgp, the expression **allocrite** was coined (39). Allocrites thus carry at least one HBA. The definitions in their simplest form are summarized in Table 1.

**Table 1:** Rules for predicting modulators, substrates, inhibitors and inducers.

**Type of allocrite**	**Patterns determining interaction with Pgp**	**Cross-sectional area determining diffusion rates^b^**
Modulator	Patterns ≥ 1 type I or type II^a^	A_D_ < 70 Å^2^, MM < 450 g/mol
Inhibitor	Patterns ≥ type I or type II	A_D_ no limit
Substrate	Patterns ≥ 1 type I or type II	A_D_ > 70 Å^2^, MM > 450 g/mol
Inducer^c^	Patterns ≥ 1 type II	A_D_ no limit

a *Hydrogen bond acceptor, i.e. π-electron donor patterns, formed by the following groups: >C=O, -O-, -NR_3_ (tertiary amino groups, but not secondary and primary amino groups), -N=, -S-, R-F, >C(C_6_H_5_), are required for an interaction with Pgp (46). π-electron systems such as phenyl residues can act as hydrogen bond acceptors and can moreover undergo π-π stacking interactions*.

b *The parameters that slow down passive diffusion are most importantly the cross-sectional area, A_D_, (or the MM) and the charge (pK_a_ value) of the drug (34, 65). Hydrophilic groups that do not interact with Pgp including -OH, >NH, -NH_2_ groups also slow down passive diffusion (48)*.

c *Induction e.g., via pregnane X receptor (47)*.

*Pgp allocrites are amphiphilic and either electrically neutral or cationic*.

## Pgp in Absorption and Excretion

Pgp is a “housekeeping” protein, expressed in many tissues of the healthy human body. It is expressed at the apical surface of the epithelia in the gastrointestinal tract and prevents drug and toxin uptake into the blood capillaries. It is thus a primary factor limiting the bioavailability of many orally administered drugs, including oral chemotherapeutic agents (73). Pgp not only limits toxin and drug absorption in the gastrointestinal tract, but most importantly, also facilitates their elimination from the intestine, the liver, kidney (74). Moreover, it reduces uptake of xenobiotics from blood into the brain at the blood-brain barrier (BBB) (i.e., endothelial cells of the blood vessels in the central nervous system) (75). Expression of Pgp is also detected in the adrenal gland, the pregnant uterus, the placental trophoblasts, the testes, and hematopoietic stem cells [for review see (76)]. Pgp is moreover present in intracellular membranes such as the endoplasmic reticulum (77) and the nuclear envelope (78).

Valuable information on the impact of total Pgp inhibition can be inferred from experiments with *mdr1* knock-out mice (79). Under culture conditions, knock-out mice exhibited normal viability, but showed altered pharmacokinetics, which had a profound effect on the tissue distribution and especially the brain accumulation of drugs. As demonstrated accidentally, a treatment with ivermectin, a pesticide to counteract a mite infestation, also used against river blindness in humans, was well tolerated by wild type mice, but turned out to be fatal for the *mdr1* knock-out littermates. In the absence of Pgp, the highly neurotoxic ivermectin was no longer prevented from reaching the central nervous system (80).

In cancer treatment, a therapeutic window for Pgp inhibition exists only if the expression level of Pgp in tumors is not higher than the expression level in the other tissues (27, 29). To find the concentration range of inhibitors tolerated by an individual (or to assess the therapeutic window), ideally, the level of Pgp in the different tissues should be measured, under clinical conditions. However, this is technically demanding and often limited by sensitivity issues as e.g., in the case of ^19^F NMR (nuclear magnetic resonance), with fluorinated drugs (81).

The inter-individual variability of Pgp expression is moreover high and depends on diverse factors including gender (82), ethnicity, age, and body mass index (BMI) (83). The Pgp expression level can additionally vary as a function of time with X-ray irradiation (84), ultra violet light irradiation (85), heat shock (86), and importantly, with diet, medication, metabolism and disease state (as discussed below).

Thus, systemic inhibition of Pgp without careful assessment of its feasibility is not advisable, even if the compounds used to “inhibit” Pgp are *per se* non-toxic. Pgp inhibition nevertheless remains an important issue, because many cancer drugs may act as Pgp inhibitors. Moreover, many therapeutic regimens include several drugs that together lead to Pgp inhibition. The phenomenon of so-called drug-drug interactions can be significant in drug regimens for cancer therapy.

## Pgp in Immunosurveillance

In the absence of external manipulations, the immune system protects the host against oncogenesis and controls the immunological features of developing tumors (87). As outlined by Zitvogel et al. (87), this process, called cancer immunoediting, consists of three phases: “first, the elimination of malignant cells by the immune system; second, the establishment of an equilibrium between genetically unstable malignant cells and the immune system, which reflects the immunoediting imposed by the immune system on cancer cells; and third, the escape of neoplastic cell variants with reduced immunogenicity, which ultimately form clinically manifest neoplasms.” Clinically significant resistance occurs only when the pre-existing resistant phenotypes are able to proliferate extensively, a process governed by eco-evolutionary dynamics (1, 88).

Mounting evidence indicates that Pgp has important functions in cancer immunosurveillance (89–94). Although, Pgp's role in immunity has long been realized [e.g., (95)], it is not fully understood, and has so far mostly been ignored in classical chemotherapy. Pgp is expressed in different cell types of the *innate immune system* [including macrophages, dendritic (DCs), and natural killer (NK) cells] as well as in different cell types of the *adaptive* immune system [including lymphocytes, bone marrow (B), and importantly thymus (T) cells (CD4+ and CD8+ cells)] (96).

In many cell types of the immune system, Pgp is co-localized with pattern recognition receptors (PRRs), including toll-like receptors (TLRs). PRRs are single-pass membrane-spanning receptors that recognize structurally conserved molecules, stemming from microbes, including nucleotides, and lipids. TLRs moreover, recognize certain drugs (97) carrying the same recognition patterns as allocrites for Pgp (Figure 2) (46). Pgp and PRRs, thus show partially overlapping substrate specificity. They seem however to differ with respect to their charge preferences. Whereas, Pgp binds cationic and electrically neutral compounds, including nucleosides, PRRs rather recognize anionic nucleotides and are strongly activated by the bacterial lipid A, flanked by anionic phosphate groups. Together with Pgp, a pattern recognition transporter (PRT), PRRs constitute an effective defense system against compounds carrying characteristic “danger” patterns that could interfere with the genetic information of cells (46).

Pgp is involved in the excretion of inflammatory cytokines from T cells or T lymphocytes (TCs) (89, 98) and DCs (73, 91). The cytokines extruded are the tumor necrosis factor (TNF)-α and INF-γ, which both play key roles in antiviral effects and immunosurveillance of cancer (87). Whether Pgp directly extrudes cytokines, or whether it rather extrudes some other physiologically relevant substrates that indirectly stimulate cytokine secretion, is not yet fully clarified. It is therefore of interest to know how the function of immune cells carrying Pgp is influenced by drugs which are also transported by Pgp.

Experiments with human alloimmune TCs *in vitro* revealed that inhibition of Pgp with tamoxifen reduced TNF-α and IFN-γ by ~80% (89). Pgp inhibition with R-verapamil in mice also significantly reduced the serum levels of TNF-α and IFN-γ, and enhanced the level of interleukin-6 (IL-6) (98). Elevated concentrations of IL-6 may indicate an ongoing inflammatory response, observed during the chemotherapy of cancers (99) or viral infections (100). Infection of the monocytic cell line THP-1with the bacterial pathogen *Listeria monocytogenes* transcriptionally induced Pgp, which activated a Type I IFN-response against *L. monocytogenes* bacteria. Both, inhibition of Pgp function by verapamil, or inhibition of its transcription using mRNA silencing, led to a reduced Type I response (i.e., reduced IFN release) in infected cells (101).

Drug-induced high levels of IL-6 correlate with high levels of the INFγ-inducible immunosuppressive ligand PD-L1 (102) and high levels of Pgp (103). PD-L1 on tumor cells interacts with PD-1 on T-cells leads to immune evasion, PD-L1 represents a novel biomarker for immune checkpoint blockade therapy (102, 104, 105).

Thus, Pgp modulators and inhibitors, including verapamil, glucocorticoids, cytostatics, clacineurin inhibitors, TOR-inhibitors (106) and many more (107) dampen or dysregulate the immune response. For comparison, mice deficient in mdr1a, spontaneously develop colitis (108) and *mdr2*-knockout mice, develop inflammation-associated hepatocellular carcinoma (109, 110).

Together, these findings demonstrate that therapeutic strategies that involve Pgp inhibition dampen or dysregulate immune reactions and may even lead to immune evasion. Thus, Pgp transporter capacity is essential for proper development of a balanced immune response by TCs and DCs against cancer cells.

## Pgp and Cancer Metabolism

Homeostasis of cellular metabolism is vital to maintaining a balanced physiological activity. A critical part of metabolism is oxidative phosphorylation, a process that uses oxygen to produce ATP in mitochondria. Cells flooded with cytotoxic drugs, try to minimize this burden by overexpressing Pgp e.g., via the PXR pathway that induces MDR (47). As extrusion of a single drug molecule by Pgp requires hydrolysis of at least one molecule of ATP (58), MDR significantly enhances the cellular ATP requirements. High rates of oxidative phosphorylation may, produce reactive oxygen species (ROS) as a byproduct, which causes oxidative damage, mutagenesis, and may initiate cancer. ROS development is particularly high, if cells are exposed either to very low or very high glucose levels (111).

Cells strongly overexpressing Pgp may become hypersensitive or collaterally sensitive to modulators such as verapamil (112) or lovastatin (113), if applied at maximum activity over an extended period of time which leads to cell death. Interestingly, hypersensitivity was attenuated if higher, inhibitory concentrations were applied, where ATPase activity (and thus ROS production) decreased again (112). Inhibitors of Pgp ATPase activity such as PSC 833 and ivermectin also reduced hypersensitivity to verapamil in MDR cells. Thus, apoptosis in resistant cell lines was likely mediated by ROS, produced in response to the high ATP activity by Pgp.

The high cellular oxygen requirement to drive Pgp may locally reduce the oxygen tension, which stimulates expression of the redox-sensitive transcription factor hypoxia-inducible factor-1 (HIF-1α). HIF-1α directly binds to the *MDR gene promotor* region inducing glycolysis (114) and further upregulats Pgp (115) to remove oxidized molecules and particularly oxidized nucleosides (46). HIF-1α thus acts as a cellular antioxidant defense (AOD) mechanism and attenuates ROS production. Thus, similar to healthy cells, cancer cells allocate significant energy to maintaining the intracellular redox balance (66) as illustrated in Figure 4.

The shift of cancer cells to glycolysis was discovered by Warburg (116). However, in contrast to early assumptions, cancer cells retain the capacity for oxidative phosphorylation and can in principle still consume oxygen at a rate similar to that of normal cells (117–119). As oxidative stress is generally transient (120) and oxidative stress and glycolysis seem mutually exclusive, we assume that the two effects are both transient and alternate.

Moreover, glycolysis may enhance the MDR phenotype of cancer cells, in a Pgp-independent manner, with consequences for the lipid bilayer membrane. Glycolysis slightly acidifies the extracellular medium and renders the cytoplasm slightly more basic (121). Under normal conditions the outer leaflet of the lipid membrane is composed of lipids with zwitterionic POPC and cholesterol, whereas the inner leaflet is composed of lipids with the zwitterionic phosphatidyl ethanolamine (PE) and negatively charged phosphatidyl serine (PS) head groups, respectively. Due to the more basic cytoplasm, PE (with pK_a_ 9.8 ± 0.1) may partially deprotonate, which allows for PE scrambling and a loss of strict membrane asymmetry. Because the head group of PE is smaller than the head group of PC, the lateral lipid packing density of the outer leaflet increases, which leads to reduced partitioning of drugs into the outer membrane leaflet, enhancing the “MDR phenotype” independent of Pgp.

**Figure 5:**
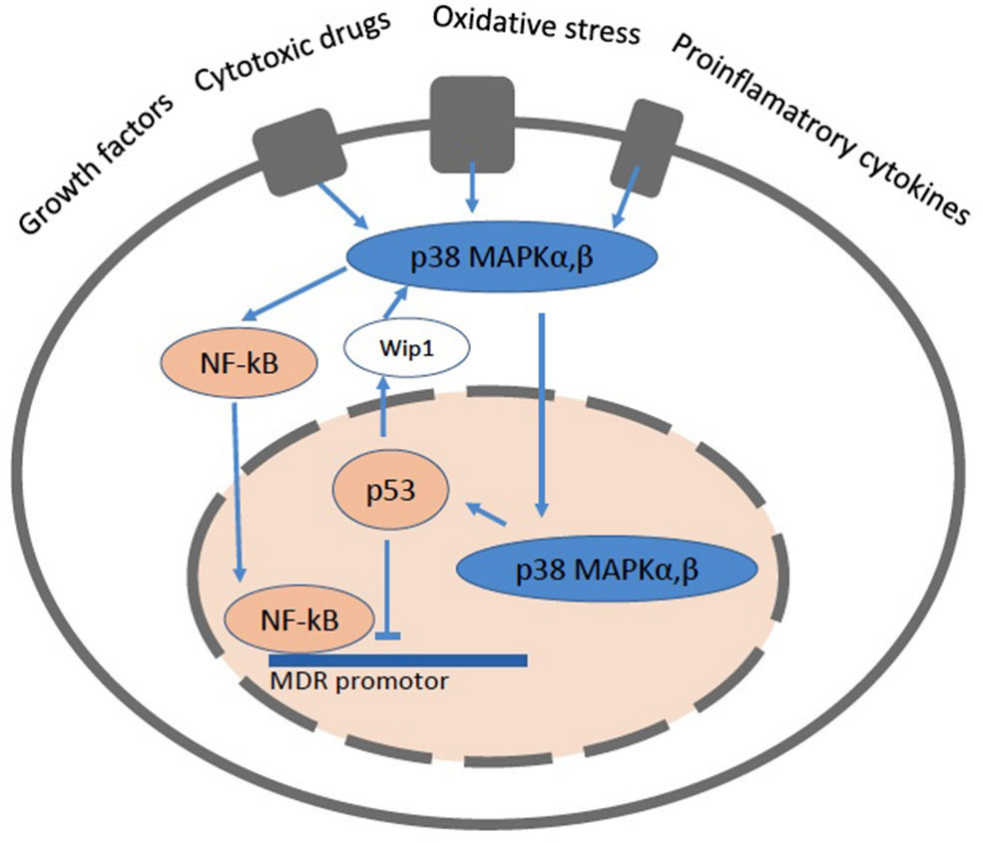
A cartoon showing a cell with the pathways related to NF-κB and p53. The mitogen activated protein kinase (MAPK) signaling pathways up-regulate the expression of Pgp via NF-κB and protect it from degradation. NF-κB can directly bind to the MDR promotor. The p38 MAPK pathway mediates cell death, cell differentiation and cell cycle checkpoints. It is activated in response to oxidative stress, cytokines and DNA damage. p38 MAPK is primarily located the cytoplasm, but upon stimuli that induce DNA double strand breaks it enters the nucleus. The p53 protein is involved in cell cycle control, apoptosis, and lipid catabolism.

The *mdr1/MDR1* gene promotor region contains numerous transcription factor binding sequences. The forkhead transcription factors O1 (FOXO1 and FOXO3a) which, induced by the silent information regulator 1 (SIRT1), enhance the *mdr1/MDR1* promotor activity. The nuclear factor-kappaB (NF-κB) (122) also enhances the *mdr1/MDR1* promotor activity. NF-κB is kept silent in the cytoplasm via interaction with the inhibitory protein IκBα and transmigrates into the nucleus upon activation. Constitutively active NF-κB has been found in the nucleus of some cancer cells. NF-κB can also interfere with p53 transcriptional activity through the competition for cofactors, which constitutes a second potential mechanism for the NF-κB antiapoptotic effect (122) (Figure 5).

A long-established link exists between p53 and the *Mdr1* gene promoter (123–125). The p53 protein is involved in cell cycle control, apoptosis, and lipid catabolism. Whereas p53 down regulates Pgp, the mutated forms of p53 are no longer able to induce apoptosis and enhance Pgp activity and lipid anabolism, which leads to tumor growth (126, 127). Consistent with these findings a p53-null mouse model revealed elevated Pgp expression in kidney, spleen and testis, suggesting a tissue-specific regulation of Pgp (128, 129). More recently it was shown that not only insufficient, but also excessive p53 expression has deleterious effects and results in cell death, lipid accumulation, inflammation and compromised tissue functionality (129).

If cells with a metabolic “deficit” undergo apoptosis induced by p53, Pgp is no longer required and is therefore down regulated. However, if apoptosis does not occur, as in cells carrying p53 mutants, Pgp continues working to eliminate oxidative waste and might be required also for lipid metabolism.

## FDA Approved Drugs Repurposed as NF-κB Inhibitors and Their Interactions With Pgp

Trial and error is still the most common procedure in cancer drug application. To improve prediction of resistance development in cancer therapy the physiological regulation of Pgp (ABCB1) and other ABC transporters such as MRPs (ABCCs) and BCRP (ABCG2), with partially overlapping substrate specificities (16–18) need to be considered in more detail. Here we suggest a set of simple rules (Table 1) to predict for any drug, based on its chemical structure, whether and how it interacts with Pgp.

**Figure 6:**
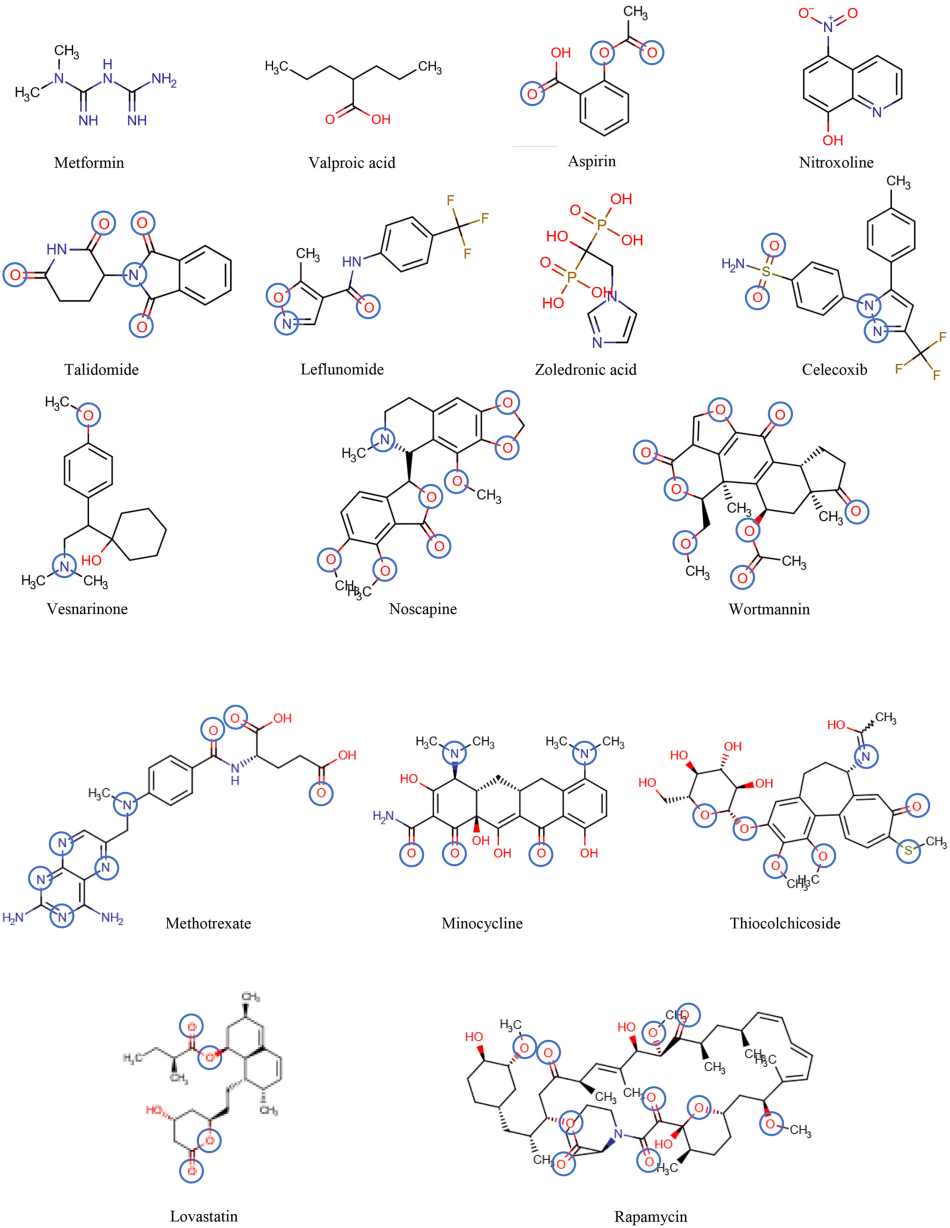
The chemical structures of the drugs listed in Table 2 are shown in the order of increasing molecular mass and hydrogen bond acceptor groups are highlighted in blue.

For proof of principle we use a set of known, FDA approved drugs, previously repurposed for cancer therapy (30) (Table 2, Figure 6). These drugs, downregulate the nuclear factor-κB (NF-κB). As NF-κB plays a crucial role in various biological processes, including immune response, inflammation, cell growth and survival, and development of malignant tumors, inhibiting NF-κB signaling has potential therapeutic applications in cancer and inflammatory diseases (146). Downregulation of NF-κB should in particular enhance apoptosis by p53 (147). Although NF-κB inhibition could be beneficial in treating inflammatory diseases and cancer, questions regarding the balance between efficacy and safety need to be considered since NF-κB function is required for maintaining normal immune responses and cell survival [for detailed information see review (148)].

**Table 2:** FDA approved non-cancer drugs repurposed for cancer treatment analyzed for their interactions with P-glycoprotein.

**Drug**	**Original indication, mechanism**	**New anticancer indication, mechanism**	**Mol mass (g/mol)**	**pK_a_**	**LogP**	**Pgp interactions predicted (see Table 1)**	**Pgp interactions measured**
Metformin	Diabetes Mellitus AMPK ↑^a^	Breast, adenocarcinoma, prostate, colorectal AMPK ↑, NF-κB ↓, TNF ↓, MCP-1 ↓	129.2	12.3	−0.92	-NR_3_ No type I/ II units No allocrite No substrate	(130, 131) No substrate
Valproic acid	Antiepileptic GABA ↑	Leukemia, solid tumors HDACI ↓, HDACII ↓, NF-κB ↓, IL-6 ↓	144.2	5.14	2.8	No type I/ II units No allocrite No substrate	(132) No substrate
Aspirin	Analgesic, antipyretic COX-1 ↓, COX-2 ↓	Colorectal cancer Prostate cancer COX-2 ↓, NF-κB ↓, AP-1 ↓	180.2	3.5	1.19	1 type II unit Allocrite/modulator Inducer	(133, 134) (135) Inducer^b^
Nitroxoline	Antibiotic	Bladder, breast cancer MetAP-2 ↓	190.2	6.88	1.99	No type I/ II units No allocrite	–
Thalidomide	Antiemetic in pregnancy TNF-α ↓	Multiple myeloma NF-κB ↓, STAT3 ↓	258.2	11.59	0.33	-NR_3_ 2 type II units Allocrite/modulator Inducer	(136) Inducer^d^
Leflunomide	Rhematoid arthritis DHODH ↓	Prostate Cancer PDGFR ↓, EGFR ↓, FGFR ↓, NF-κB ↓	270.2	10.8	2.8	-NR_3_ Type II unit Allocrite Inducer	(137) Interaction with BCRP^c^
Zoledronic acid	Anti-bone resorption Osteoclast ↓	Multiple myeloma, prostate cancer, breast cancer CXCR-4 ↓, MMPs ↓, IL-6 ↓, Bcl-2 ↓, Bax ↑, FOXO3α ↑	272.0	0.7, 6.7		No allocrite	–
Celecoxib	Osteoarthritis, rheumatoid arthritis COX-2 ↓	Colorectal cancer, lung cancer COX-2 ↓, NF-κB ↓	381.4	11.1	3.53	-NR_3_, 1 type I unit Allocrite	(138) Pgp repression^b^
Vesnarinone	Cardioprotective	Oral cancer, leukemia, lymphoma NF-κB ↓, IL-8 ↓, VEGF ↓, AP-1 ↓	395.5	2.86		-NR_3_, 1type I unit Allocrite/modulator	(139) Inhibitor^e^
Statins, e.g., Lovastatin	Myocardial infarction HMG-CoA reductase ↓	Prostate cancer, leukemia NF-κB ↓, HMG-CoA reductase ↓	404.5	4.26	~4	2 type I units Allocrite/modulator	(113) Modulator^f^
Noscapine	Antitussive, antimalarial, analgesic Bradykinin ↓	Multiple cancer types NF-κB ↓, HIF-1α ↓, Bcl-2 ↓, p21 ↑, p53 ↑, AIF ↑	413.4	12.86 7.14	2.85	-NR_3_, 2 type I, 1 type II units Allocrite Inhibitor Inducer	(140) Inhibitor
Wortmannin	Antifungal	Leukemia NF-κB ↓, AP-1 ↓	428.1	–	–	Type I / II units Non-amphiphilic MRP2 substrate	(141) MRP2 substrate
Methotrexate	Acute leukemia DHFR ↓	Osteosarcoma, breast cancer, Hodgkin lymphoma NF-κB ↓, TNF-α ↓	454.2	4.8, 5.5	0.74	-NR_3_, 2 type I or 1 type II Substrate	(142) Substrate^g^
Minocycline	Acne	Ovarian cancer, glioma MMPs ↓	457.5 g	–	−0.03	-NR_3_, 1 type II unit Substrate Inhibitor Inducer	(143) Substrate Inhibitor
Thio-colchicoside	Muscle relaxant GABA ↓	Leukemia, multiple myeloma NF-κB ↓	563.2	12.74	0.34	-NR_3_, Type I/II units Substrate Inhibitor Inducer	(144)
Rapamycin	Immunosuppressant mTOR ↓	Colorectal cancer, lymphoma, leukemia NF-κB ↓, IL-6 ↓, IKK ↓	914.19	~9	4.3	-NR_3_, Type I / II units Substrate Inhibitor	(145) Substrate Inhibitor

a *AMPK, AMP-activated protein kinase; AIF, apoptosis-inducing factor; AP-1; Bax, Bcl-2-associated X protein; Bcl-2; BCRP, breast cancer resistance protein; CXCR-4, CXC chemokine receptor-4; DHFR, dihydrofolate reductase; DHODH, dihydroorotate dehydrogenase; FGFR, fibroblast growth factor receptor; FOXO, forkhead homeobox type O; GABA, γ-aminobutyric acid; HIF-1α, hypoxia-inducible factor-1α; HMG-CoA; IKK; MCP-1, monocyte chemoattractant protein-1; MetAP, methionine aminopeptidase; MMP, matrix metalloproteinase; mTOR; NF-κB; p21; p53; VEGF; ↑, upregulation; ↓, downregulation*.

b *Conflicting data may be due the fact that Aspirin is a Pgp inducer [see also (136)] and possibly may at the same time reduce Pgp expression via COX-2 inhibition. The same is true for celecoxib. Specific COX-2 inhibition may be used as a new therapeutic strategy to prevent seizure-induced P-glycoprotein up-regulation at the blood-brain barrier (138)*.

c *Pgp and BCRP have overlapping substrate specificity (16, 18)*.

d *Treatment with thalidomide produced a concentration- and time-dependent induction of Pgp expression in rat trophoblasts. By contrast, in human trophoblasts, thalidomide decreased the expression of Pgp in a concentration- and time-dependent manner. The difference of trophoblast behavior between both culture models, i.e., rat and human is also noted in vivo for the teratogenic effect of thalidomide between rat and human*.

e *Most allocrites can be inhibitors at high concentrations*.

f *P-glycoprotein expressing cells exhibited a collateral sensitivity to lovastatin. Collateral sensitivity occurs when allocrites strongly activates the Pgp ATPase activity and produce ROS, see e.g., Verapamil (112)*.

g *Thus, a deficiency in the methotrexate (MTX) carrier enables Pgp to confer resistance to MTX, suggesting that hydrophilic compounds become Pgp substrates when they enter cells by passive diffusion*.

*The table is adapted from Gupta et al. (30)*.

Table 2 includes 16 drugs (column 1), the original indication with mechanisms (column 2), and the new antitumor indications with mechanisms (column 3) (30). To describe the drugs from the physical chemical point of view, the molecular mass, the pK_a_ value (for charge estimation) and LogP value (for lipophilicity) (columns 4, 5 and 6) [see (65)]. The number and type of recognition patterns per drug for the interaction with Pgp (column 7) and the predictions of whether or not the drug is an allocrite, modulator, substrate, inhibitor or inducer, assessed according to rules in Table 1, are given in column 8 and can be compared with the experimental data in the literature (column 9). An excellent agreement between the predicted behavior of the drugs (column 8) and the experimental Pgp data (column 9) proves that interactions of drugs with Pgp can be reliably predicted on the basis of this broad physical chemical analysis. Further confirmation for the developed approach came from *in silico* predictions based on large data sets, e.g., (133, 149). These simulations come to similar conclusions regarding relevant groups for the overall interactions of drugs with Pgp, however, without any insight into the binding mechanism (i.e., differentiation between partitioning into the membrane and binding proper to Pgp within the lipid membrane that determine overall Pgp binding).

## Conclusions

We demonstrate that Pgp, unlike other transporters, captures drugs in the lipid membrane by attracting them via weak electrostatic interactions (including hydrogen bonding, π-π stacking, and π-cation interactions). The interactions are strong in the lipid membrane and are overcome as soon as the polar part of the amphiphilic drugs sense water at the membrane-water interface. We further demonstrate that Pgp is a *pattern recognition transporter* that shows overlapping substrate specificity with *pattern recognition receptors* (PRRs) (section The Floppase Model and the Consequences for Drug Pgp Interactions). Pgp gets inhibited or, more accurately, slowed down, when its cargo is large. This happens more often than expected, particularly when complex drug regimens are applied, because Pgp can simultaneously accommodate different drug molecules (section What Defines a Pgp Modulator, Inhibitor, Substrate, Inducer and Allocrite?). Inhibiting Pgp bears the danger of intoxication due to enhanced drug uptake and reduced metabolite excretion (section Pgp in Absorption and Excretion). Moreover, it leads to immunosuppression and finally immune evasion which is mostly overlooked as yet (section Pgp in Immunosurveillance).

Healthy and cancer cells try to maintain homeostasis. However, this is challenging in the presence of cytostatic drugs. To remove toxins, cells upregulate Pgp that consumes ATP. Enhanced oxidative phosphorylation to provide sufficient ATP, causes reactive oxygen species (ROS). ROS in turn damage cells by causing oxidative debris or oxidized nucleosides that are also cytotoxic. To counteract this phenomenon and to reduce oxidative stress, cells switch to glycolysis via HIF-1α and again upregulate Pgp for cleaning up (section Pgp and Cancer Metabolism). We finally show that simple rules (derived from the quantitative thermodynamic and kinetic analyses of Pgp-drug interactions) allow predicting whether a drug will be a Pgp ATPase modulator or inhibitor, will be exported by Pgp or will further induce Pgp. As a proof of concept we apply these rules to analyze a set of FDA approved drugs, repurposed for cancer therapy (30). An excellent agreement between predictions and published experiments is obtained. These predictions may help to improve treatment regimens.

As an aside, trying to kill multi-resistant bacteria on a systemic basis using an antibacterial cocktail to inhibit efflux transporters over a short period of time may be feasible. However, using Pgp inhibitors or combinations of several drugs that together inhibit Pgp over longer periods of time is not curative or life prolonging. The complexity of cancer metabolism could here only be touched on and more effort will be required to characterize the individual cancer types and find ways to subtly shift homeostasis back to states that approach “healthy” conditions.

## Author Contributions

The author confirms being the sole contributor of this work and has approved it for publication.

## Conflict of Interest

The author declares that the research was conducted in the absence of any commercial or financial relationships that could be construed as a potential conflict of interest.

## Abbreviations

AIF: Apoptosis inducing factor
AMPK: AMP-activated kinase
AP-1: Activator protein 1 (transcription factor)
Bax: Apoptosis regulator, bcl-2-like protein 4
Bcl-2: Bcl-2 (B-cell lymphoma 2)
COX-1, COX-2: Cyclooxygenase
DHFR: Dihydrofolate reductase
DHODH: Dihydroorotate dehydrogenase
EGFR: estimated glomerular filtration rate
FOXO3α: Forkhead transcription factors
GABA: γ-Aminobutyric acid
HDAC I, HDAC 2: Histone deacetylase
HIF-1α: Hypoxia-inducible factor 1-alpha
HMG-CoA: β-Hydroxy β-methylglutaryl-CoA
IKK: IκB (kinase master regulator of NF-κB signaling)
IL-6: Interleukin-6
IL-8: Interleukin-8
MCP-1: Monocyte chemoattractant protein 1 (cytokine)
MetAP-2: Methionine aminopeptidase 2
MMPs: Matrix metallopeptidases
mTOR: Mammalian target of rapamycin
NF-κB: Nuclear factor κ-light-chain-enhancer of activated B cells
p21: Cyclin-dependent kinase inhibitor (CKI)
p53: Tumor-suppressor gene (TSG)
PDGFR: Platelet-derived growth factor
STAT 3: Signal transducer and activator of transcription 3
TNFα: Tumor necrosis factor (cytokine)
VEGF: Vascular endothelial growth factor.
